# Biochemical insights and microRNA profiling via next-generation sequencing in moderate and severe COVID-19 cases

**DOI:** 10.1016/j.clinsp.2026.101018

**Published:** 2026-06-10

**Authors:** Mahnoor Khan, Awais Altaf, Naeem Ashraf, Syed Zeeshan Haider Naqvi, Hafiz Muhammad Hammad, Tahir Maqbool, Tariq Aziz, Maha Abdullah Alwaili, Rania Ali El Hadi Mohamed, Maher S. Alwethaynani, Fakhria A. Al-Joufi, Deema Fallatah

**Affiliations:** a Institute of Molecular Biology and Biotechnology, The University of Lahore, Lahore, Pakistan; b Federal Postgraduate Medical Institute, Shaikh Zayed Hospital, Lahore, Pakistan; c Faculty of Health Sciences, Equator University of Science and Technology, Masaka, Uganda; d School of Biochemistry and Biotechnology, University of the Punjab, Lahore, Pakistan; e Biodiversity Genomics Unit, Faculty of Science, University of Tabuk, 71491, Tabuk, Saudi Arabia; f Department of Biology, College of Science, Princess Nourah bint Abdulrahman University, P.O. Box 84428, Riyadh, 11671, Saudi Arabia; g Department of Clinical Laboratory Sciences, College of Applied Medical Sciences, Shaqra University, Alquwayiyah, Riyadh, Saudi Arabia; h Department of Medical Laboratory Sciences, College of Applied Medical Sciences, Prince Sattam bin Abdulaziz University, Saudi Arabia; i Department of Pharmacology, College of Pharmacy, Jouf University, Saudi Arabia

**Keywords:** SARS-CoV-2, microRNA, microRNA Differential expression, NGS, NF-κB Pathway

## Abstract

COVID-19 remains the most devastating and challenging viral disease in human history

**Objective:**

This study explores the biochemical and host microRNA (miRNA) response to Severe Acute Respiratory Syndrome Coronavirus-2 (SARS-CoV-2) infection and explores how DE miRNAs alter molecular pathways to identify potential prognostic and therapeutic biomarkers.

**Methods:**

Biochemical profiling on automated chemistry analyzers and circulating miRNA profiling by Next Generation Sequencing were performed in moderate (ward) and severe (ICU) patients of COVID-19. miRNA Differential Expression (DE) was analyzed in ICU vs. Control and Ward vs. Control Groups. Statistical comparison of biochemical variables amongst ICU, Ward, and Control groups was done on SPSS by applying the Kruskal-Wallis test; p*-*value < 0.05 was considered significant. miRNA DE analysis was performed with the help of a bioinformatics tool, DESeq2. A False Discovery Rate (FDR) adjusted p-value < 0.05 was considered to be significant.

**Results:**

Most biochemical parameters were significantly and proportionately altered with respect to the severity of the disease. Patients showed compromised renal, hepatic, and cardiac function, as well as deranged blood pictures and elevated inflammatory markers (CRP, IL-6, LDH, and Ferritin). miRNA Differential Expression (DE) when analyzed in both study groups, 102 DE miRNAs were found in the ICU vs. Control group, of which miR-423–5p was significantly upregulated, and miR-744–5p was significantly downregulated. Whereas, in the Ward vs. control group, out of 91 DE miRNAs, only miR-486–5p was observed to be significantly upregulated. Gene targets of these three miRNAs were further explored along with their cellular pathways. Literature suggests that these affected cellular pathways converge on the stimulation of the NF-κB pathway. Biochemical evaluation of COVID-19 cases reveals major disease severity predictors like raised inflammatory markers CRP, IL-6, LDH, elevated organ function tests and deranged hematological profiles.

**Conclusion:**

Three differentially expressed miRNAs were observed that might be involved in the activation of NF-κB, which acts as a major modulator of inflammation in response to SARS-CoV-2 infection. The miRNA findings are preliminary and hypothesis-generating, only requiring independent validation e.g., qPCR in a larger cohort. The probable theragnostic potential of these miRNAs may be clinically explored further by the use of miRNA mimics or miRNA inhibitors that may change the adversity associated with this virus altogether.

## Highlights

• COVID-19 clinical profiling identifies biochemical markers of disease severity.
• NGS miRNA profiling identifies dysregulated miRNAs in severe COVID-19.
• miRNA profiling reveals insights into COVID-19 pathogenesis and therapy.

## Introduction

SARS-CoV-2 is a highly contagious virus that emerged in Wuhan, China, in 2019 and engulfed the whole world with the devastating disease named Coronavirus Disease 2019 or COVID-19. It spread very quickly to other parts of the world, and the magnitude of the catastrophe rose to more than 6 million deaths globally. The fast spread of this virus, along with the associated high rate of morbidity and mortality, compelled the World Health Organization to label it as a pandemic in March 2020.^1^^,^^2^

The genome of SARS-CoV-2 comprises a single positive RNA strand, almost 30 kbp in length. Major structural proteins of this virus include Spike glycoprotein (S), Membrane protein (M), Nucleocapsid protein (N), and Envelope protein (E). Two major subunits of spike protein, S1 and S2, have the ability to bind to the host Angiotensin-Converting Enzyme-2 (ACE-2) and enter into the host cell.^3^

A wide spectrum of clinical outcomes emerged after its exposure, ranging from a patient being completely asymptomatic to a patient with a life-threatening condition characterized by Acute Respiratory Distress Syndrome (ARDS) and multiple organ failure. Common symptoms include cough, fever, dyspnea, diarrhea, fatigue, and pneumonia.^4^ COVID-19 is therefore classified into mild, moderate, severe, and critical forms of presentation owing to age, immune status, and comorbidities. Mild and moderate forms of the disease present with upper respiratory tract involvement without dyspnea or pneumonia. Whereas, severe and critical COVID-19 patients present with shortness of breath, pneumonia, and ARDS.^5^ Biochemical and hematological evaluation of COVID-19 patients hence, plays a critical role in early diagnosis and effective management of the disease.^6^^,^^7^

SARS-CoV-2 infection initiates an intense inflammatory reaction reflected by hematological disturbances and high cytokine levels that lead to ARDS. Immune dysfunction is manifested by lymphocyte and granulocyte abnormalities, whereas raised cytokines, including Interleukin-6 (IL-6), along with C-Reactive Protein (CRP), Lactate Dehydrogenase (LDH), and Ferritin are indicators of inflammation.^8^ Blood tests thus help to assess the inflammatory status and collateral effect on other vital organs (heart, liver, and kidney), which is highly critical for a better prognosis of the disease.^9^ Elevated levels of CRP, IL-6, d-dimer, and renal dysfunction have been reported as predictors of poor prognosis in COVID-19.^10^

Moreover, circulating microRNAs (miRNAs) have also emerged as important diagnostic biomarkers in various clinical conditions, including malignancies, systemic diseases, and infections such as COVID-19.^11^^,^^12^ miRNAs are small, single-stranded, noncoding RNAs that are 18‒25 nucleotides long. They regulate mRNA gene expression at the post-transcriptional stage and significantly affect biological processes. They alter the mRNA gene expression by binding to specific miRNA Response Elements (MREs) present usually at the 3′Untranslated Regions (UTRs) of target mRNAs. This binding generally results in repression or complete degradation of target mRNAs.^13^^,^^14^ Marinini B et al. concluded in 2022 that host miRNAs play critical roles in the adaptive and innate immune responses, especially in the development of the cytokine storm, thus qualifying as potential diagnostic, prognostic and therapeutic biomarkers of SARS-CoV-2.^15^ Eight Differentially Expressed (DE) miRNAs were identified in the patients affected by SARS-CoV-2 when compared with healthy controls. Interestingly, the differences between the three of them appeared during the early phase of the disease, which reflected their higher sensitivity and greater diagnostic potential for this virus.^16^ Gonzalvo-Calvo et al. observed ten dysregulated circulating miRNAs when ward and ICU patients of COVID-19 were compared. They helped to segregate the patients based on disease severity. Additionally, miR-192–5p and miR-323a-3p clearly differentiated between survivor and non-survivor ICU patients. Hence, miRNA profiling in severely affected COVID-19 patients promises to be a significant tool for risk stratification.^17^

Reinfections with COVID-19 have been reported worldwide. It is now evident that the naturally acquired immunity after exposure does not provide protection after a few months. This may be due to a lack of effective immunity or the existence of variants causing resistance.^18^ The large number of viral hosts leads to increased replication and a higher chance of mutations with an increased rate of transmission.^19^ A latest review narrates a high level of protection against reinfections from ancestral, alpha, beta and delta variants, but very low protection against the omicron variant BA.1.^20^

Keeping in view these advancements, it is imperative to unveil the connections of genetic factors linked with the biological pathways causing the disease. Most importantly, there is still a lack of knowledge with respect to how DE host miRNAs affect pathological molecular mechanisms that alter the disease status. To address this research gap, the authors aimed at first to evaluate the biochemical markers along with circulatory miRNA gene expression in moderate (ward) and severe (ICU) COVID-19 patients. Secondly, differentially expressed miRNAs were correlated with their gene targets in an attempt to explore the underlying pathological mechanisms of this viral disease. Identification and hence comprehension of DE miRNAs and their affected molecular pathways will highlight their roles in risk stratification, early diagnosis, effective management, and avoiding the resurgence of this pandemic.

## Materials and methods

This research was conducted at the Biochemistry Department of the Institute of Molecular Biology and Biotechnology (IMBB), The University of Lahore. It was approved by the IMBB Ethical Review Committee, University of Lahore (IMBB/BBBC/22/120). All the data acquired was kept confidential. This cross-sectional study was conducted and reported in accordance with the STROBE guidelines.

### Study design

It was an observational and cross-sectional study designed for biochemical evaluation and miRNA profiling of mild and moderate COVID-19 patients by NGS.

### Sample collection

This study included 60 participants between 18–75 years of age who were recruited after receiving written and informed consent during the second phase of the COVID-19 pandemic in Pakistan (2021–22). Out of these 60 subjects, 20 were diagnosed COVID-19 patients admitted to the ward, 20 were diagnosed COVID-19 patients admitted to the ICU, and 20 were healthy controls.

Inclusion criteria were diagnosed COVID-19 patients admitted to the ward or ICU of the local hospitals of Lahore with less than two weeks of duration of the disease onset. Exclusion criteria included patients having COVID-19 for more than 2-weeks and patients with any major systemic disease or post-COVID-19 complications. This was done to capture miRNA profiles at the early stages of infection, avoiding confounding factors associated with organ dysfunction or chronic complications. These patients were recruited from the COVID-ICU and COVID-Ward of Shaikh Zayed Hospital, Lahore, and Mayo Hospital, Lahore. ICU patients and ward patients reflected the severity of COVID-19. Simultaneously, twenty healthy subjects, ten males and ten females, of the same age group with negative COVID-19 PCR tests were selected as controls. Written and informed consent was obtained from all the study participants after briefing them about the project.

Blood samples of all the subjects were collected. Blood samples for Complete Blood Count (CBC) were collected in EDTA vacutainers, whereas, for other estimations, the blood after clotting underwent centrifugation. The separated serum was kept in labelled aliquots and stored at −80 °C.^21^

### Biochemical analysis

The biochemical parameters of all the participants were measured on an automated chemistry analyzer, AU 680 Beckman & Coulter and Sysmex. Kits belonged to the Beckman & Coulter company, and all parameters were performed spectrophotometrically except Trop-I, which was performed on the chemiluminescence.^21^^,^^22^ Renal function tests, liver function tests, and cardiac troponin I were performed to assess the organ function. CRP, IL-6, LDH, and Ferritin markers were also measured to predict the extent of inflammation in these patients. In addition, other markers reported to be associated with the severity of COVID-19, including CBC and d-Dimer, were also measured. All the parameters were compared between the 3 groups: ICU, Ward, and Control group.

After biochemical analysis, out of the sixty participants, ten subjects were further selected for miRNA profiling by NGS. Only those patients were selected who had no pre-existing systemic illness or post-COVID complications. A total of eight COVID-19 patients, including four ICU patients (2 males and 2 females) and four ward patients (2 males and 2 females), along with two healthy controls (1 male and 1 female), were selected.

Nasopharyngeal swabs of healthy controls were collected from the posterior nasopharynx and removed while rotating. Total RNA was extracted, and RT-qPCR was performed using a standard SARS-CoV-2 nucleic acid detection kit (CE-IVD) according to the prescribed guidelines for the qualitative detection of SARS-CoV-2.^23^

### RNA extraction and next generation sequencing (NGS)

**Fig. 1:**
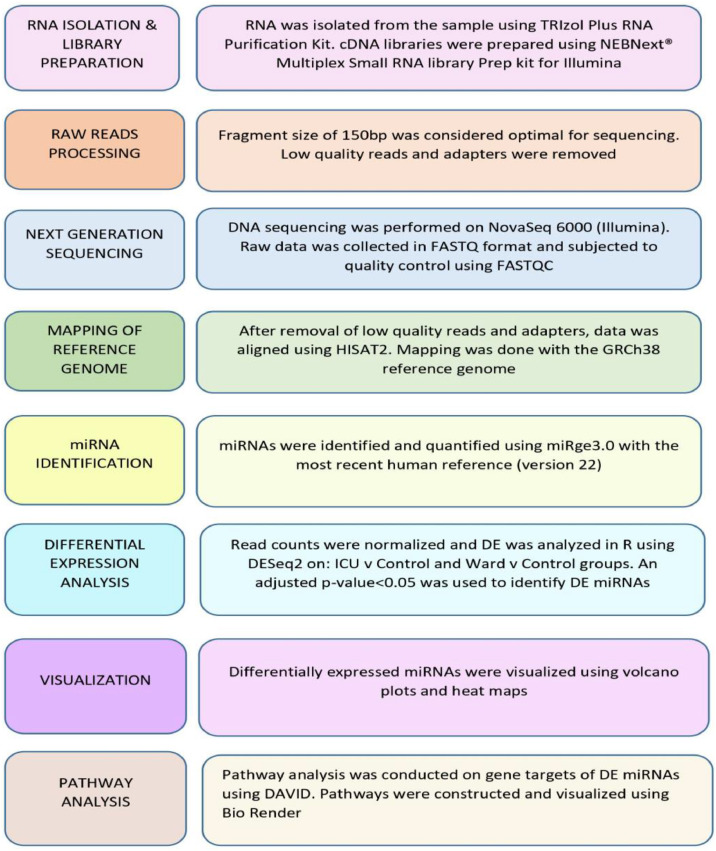
miRNA differential expression analysis by next generation sequencing.

The methodology adopted for NGS is summarized in Fig. 1. Total RNA, including small RNA, was isolated from 200 µL of a sample using the optimized protocol for the TRIzol™ Plus RNA Purification Kit. Complementary DNA libraries were prepared using the NEBNext® Multiplex Small RNA Library Prep Kit for Illumina (Index Primers 1–48, #7560S) according to the given protocol. The quality and yield after sample preparation were measured with the Fragment Analyzer. The final products were consistent with the expected size of approximately 150 base pairs.

DNA sequencing using the NovaSeq 6000 (Illumina) was performed according to the manufacturer's protocols. The prepared libraries were pooled in equimolar amounts and loaded onto the Illumina NovaSeq 6000 flow cell. Sequencing was performed in paired-end mode with 150 bp read length, aiming for a depth of 20 million reads per sample. A concentration of 1.1 nM of DNA was used. NovaSeq control software NCS v1.8 was used. Bcl files were obtained and converted into FASTQ with the Illumina data analysis pipeline RTA3.4.4 and Bcl Convert v3.10.5. Raw sequencing data were collected in FASTQ format and subjected to quality control measures using FastQC (Babraham Bioinformatics, UK) to ensure high-quality data. Any low-quality reads and adapter sequences were identified for subsequent trimming by the Trimmomatic tool.^24^ Data was aligned using the HISAT2 alignment tool.^25^ Reference genome assembly GRCh38 was downloaded from NCBI. miRNA identification and quantification were carried out using the miRge 3.0 tool^26^ using the most recent human reference miRBase (version 22). Read counts were normalized, and differential expression analysis was performed in R using the DESeq2 package version 1.30.1.^27^ An adjusted False Discovery Rate (FDR) of a p-value threshold of < 0.05 was used to identify DE miRNAs. This analysis was performed on two groups i.e., ICU vs. Controls and Ward vs. Controls. DE miRNAs were visualized in the form of heat maps and volcanoes. Target genes were explored using Target Scan and a literature search. DAVID was used for pathway analysis.^28^

### Data analysis

Statistical analysis was performed using SPSS version 20. After testing the normality of data, the non-parametric Kruskal-Wallis test was applied to compare all the biochemical parameters between the ICU, Ward, and Control groups. All the variables are expressed as median (Q1; Q3). The probability or p-value < 0.05 was considered to be statistically significant. miRNA differential expression was observed using the miRge3.0 tool that utilizes DESeq2. A False Discovery Rate (FDR) adjusted p-value < 0.05 was considered to be significant. Differentially expressed miRNAs were visualized using volcano plots and heat maps. miRNA target genes were identified using the latest version of Target Scan. An FDR value <0.05 was considered significant. A literature search was also used to identify specific gene targets. Pathway analysis was conducted on gene targets of differentially expressed miRNAs using DAVID. Pathways were constructed and visualized using BioRender.

## Results

Twenty subjects each were included in the control, ICU, and ward groups that were age and gender matched. Biochemical analysis was performed on all 60 participants of this project, whereas, owing to financial constraints, miRNA sequencing by NGS was performed only on 10 participants who were further selected considering patients who didn’t have any pre or post COVID-19 systemic conditions.

### Biochemical analysis

The biochemical parameters of all participants of the study were measured, and their results were compared among all three study groups, namely ICU, Ward, and Control groups. These parameters included renal function tests, liver function tests, inflammatory markers, complete blood count, and cardiac marker Troponin I. All the measured results, their comparison between the three groups, and their respective p-values are given in Table 1. The data of each variable is presented in the form of median (Q1; Q3).

**Table 1:** Biochemical parameters of all study groups expressed as median (Q1; Q3).

	Biochemical Parameters	ICU (n = 20)	Ward (n = 20)	Control (n = 20)	p-value
**Renal Function Tests**	BUN (mg/dL)	35.50 (26.75; 48.75)	28.50 (15.25; 45.75)	12.50 (8.25; 15.00)	<0.001
	Creatinine (mg/dL)	1.25 (0.53; 1.90)	1.10 (0.90; 1.48)	0.75 (0.53; 0.80)	0.004
**Liver Function Tests**	Total Bilirubin (mg/dL)	0.60 (0.50; 0.88)	0.50 (0.33; 0.68)	0.60 (0.50; 0.78)	0.341
	Direct Bilirubin (mg/dL)	0.20 (0.13; 0.30)	0.10 (0.10; 0.28)	0.20 (0.10; 0.20)	0.171
	ALP (U/L)	111.50 (82.50; 191.25)	97.50 (70.50; 136.75)	90.0 (77.50; 101.00)	0.291
	ALT (U/L)	48.50 (35.50; 83.00)	26.50 (15.50; 54.00)	20.0 (15.25; 25.75)	0.001
	AST (U/L)	46.50 (29.00; 88.75)	41.50 (25.75; 73.25)	24.50 (21.25; 30.50)	0.001
**Inflammatory Markers**	LDH (U/L)	695.0 (473.10; 851.43)	448.40 (321.50; 661.15)	163.50 (148.75; 180.50)	<0.001
	CRP (mg/L)	99.75 (13.65; 210.43)	28.00 (13.00; 55.20)	0.60 (0.43; 0.88)	<0.001
	IL-6 (pg/mL)	18.30 (11.38; 43.68)	3.40 (0.90; 5.25)	2.75 (1.50; 4.63)	<0.001
	Ferritin (g/L)	588.55 (61.58; 1574.75)	682.45 (382.33; 1278.81)	145.00 (95.50; 273.50)	0.006
**Complete Blood Count**	Hemoglobin (g/dL)	11.25 (10.25; 12.63)	10.75 (8.95; 13.38)	12.70 (11.90; 13.75)	0.044
	WBC × 10^3^ /µL	16.65 (11.35; 23.71)	9.80 (6.84; 12.94)	6.90 (5.88; 8.95)	<0.001
	Neutrophil %	89.85 (83.5; 93.2)	84.85 (79.40; 90.02)	50.25 (48.08; 53.08)	<0.001
	Lymphocyte %	4.67 (2.60; 10.85)	6.8 (3.88; 13.68)	35.15 (28.25; 38.75)	<0.001
	D-Dimer (µg/mL)	9.60 (3.20; 1025.50)	178.0 (32.05; 554.50)	0.49 (0.19; 0.64)	<0.001
**Cardiac Marker**	Troponin I (ng/mL)	0.05 (0.01; 0.19)	0.01 (0.00; 0.03)	0.02 (0.01; 0.03)	0.003

All biochemical markers were compared between all three study groups, and the results are presented in Table 2. Both renal function markers BUN and Creatinine were significantly elevated in both ICU and ward patients (BUN p < 0.001, Creatinine p = 0.004) as compared to healthy controls, that reflect compromised renal function. In fact, a greater degree of elevation in the ICU patients than in the ward ones suggests that it is directly proportional to the severity of the disease. Similarly, liver enzymes ALT and AST are significantly elevated (p = 0.001) in both disease groups in proportion to the severity of the disease. On the other hand, ALP, Total Bilirubin, and Direct Bilirubin do not show any significant rise (p = 0.291, 0.341, and 0.171, respectively). This reflects the initial stage of liver damage in which ALT and AST are mostly deranged.

**Table 2:** Reported Gene Targets of DE miRNAs and their associated cellular pathways implicated in COVID-19.

miRNAs	Gene Targets	Cellular pathway	Reference
miR–423–5p ↑	MALAT-1	NF-κB pathway ↑	40
	TNIP2		41
	SRF		43, 44
miR–744–5p ↓	PTBP1	NF-κB pathway ↑	45,46
	HLA-G	↓ NK cells	47
	TGF-B1	MAPK/NF-κB pathway ↑	48
miR–486–5p ↑	OTUD7B	NF-κB pathway ↑	51, 52
	FOXO1		54, 55, 57

More importantly, all the markers of inflammation and tissue damage, including LDH, CRP, and IL-6, were observed to be significantly raised (p < 0.001) in ICU and ward groups, manifesting an exaggerated inflammatory response in accordance with the severity of SARS-CoV-2. In addition, Ferritin levels also increased highly in both disease groups (p = 0.006), which is also suggestive of the acute inflammatory state of these patients.

A complete blood count reveals interesting details. Hemoglobin levels are decreased in both study groups (p = 0.04), more so in the ward group than in the ICU group. Whereas leukocyte count is significantly and proportionately increased in both ICU and ward patients (p < 0.001), reflecting increased susceptibility to infections in these patients. Significant neutrophilia (p < 0.001), as shown in both disease groups, indicates an intense inflammatory state in them. On the other hand, profound lymphocytopenia, as observed by the significantly decreased lymphocyte percentage (p ≤ 0.001), is strongly associated with viral infections. An interesting finding is a significant rise in d-Dimer levels of both disease groups (p < 0.001), with a more substantial increase in the ward patients compared to the other parameters. This reflects a higher tendency of clotting among the ward patients compared to the ICU patients, or effective prophylactic measures adopted to combat clotting issues in ICU patients of COVID-19.

Lastly, among the biochemical parameters, Troponin I levels were significantly raised (p = 0.003) in the ICU patients, reflecting a substantial impact of SARS-CoV-2 on cardiac function as well.

**Fig. 2:**
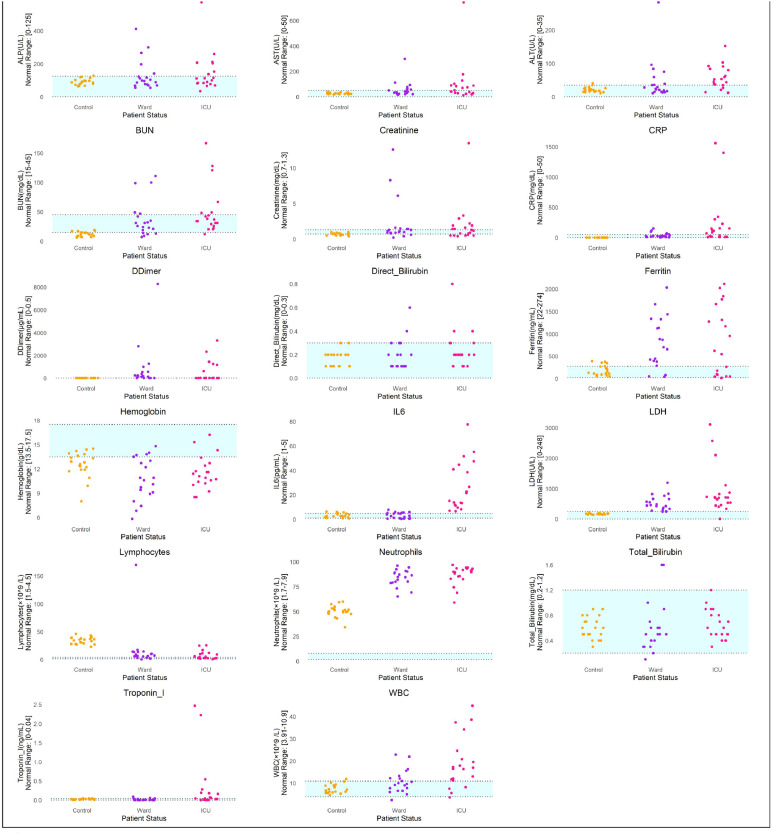
Combined scatter plots of each biochemical marker in controls, ICU and ward patients denoted by orange, purple and pink colors respectively. The highlighted area projects the normal range of each biomarker.^29.^

All the biochemical parameters in the three study groups are also given in the form of combined scatter plots in Fig. 2 below.

**Fig. 3:**
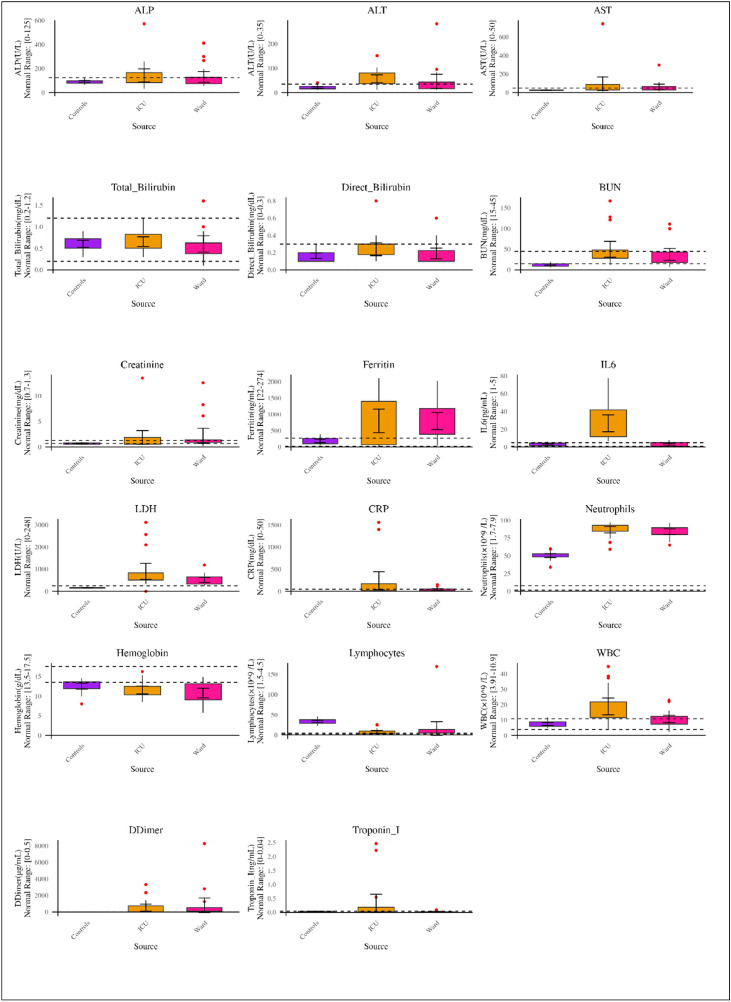
Combined box plots of each biochemical marker in controls, ICU and ward patients denoted by purple, orange and pink colors respectively.

These results are also presented in the form of box plots in Fig. 3 below.

### miRNA profiling by NGS

Raw reads obtained after NGS were submitted to the NCBI, SRA (short read archive) database (project accession n°SUB14420173). Quality check was run with FASTQC. After removing the adapters and low-quality reads by the Trimmomatic tool, HISAT2 was used for alignment and mapping with GRCh38, the human reference genome. miRBase version 22 was used for alignment by the miRge3.0 tool as a reference database to identify and quantify miRNAs. The count-based Differential Expression (DE) analysis was performed on the following two study groups: ICU vs. Control and Ward vs. Control using DESeq2 version 1.30.1. All the differentially expressed miRNAs in both these groups were identified and were further subdivided into upregulated and down-regulated miRNAs. This DE analysis was based on log2fold change and False Discovery Rate (FDR) corresponding to an adjusted p-value threshold of < 0.05 that was considered to be significant. Results were analyzed in the form of heat maps and volcano plots.

ICU vs. Control

**Fig. 4:**
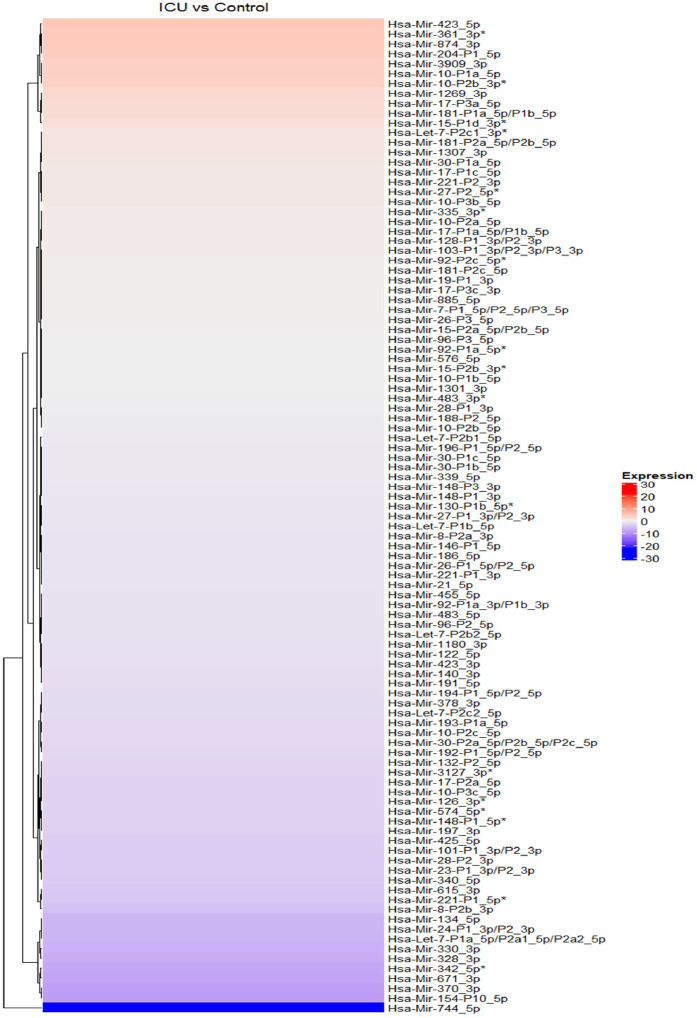
Heat map of ICU vs. Control group revealing all the DE host miRNAs in response to SARS-CoV-2 infection. The upregulated miRNAs shown in red while the downregulated ones in blue.

**Fig. 5:**
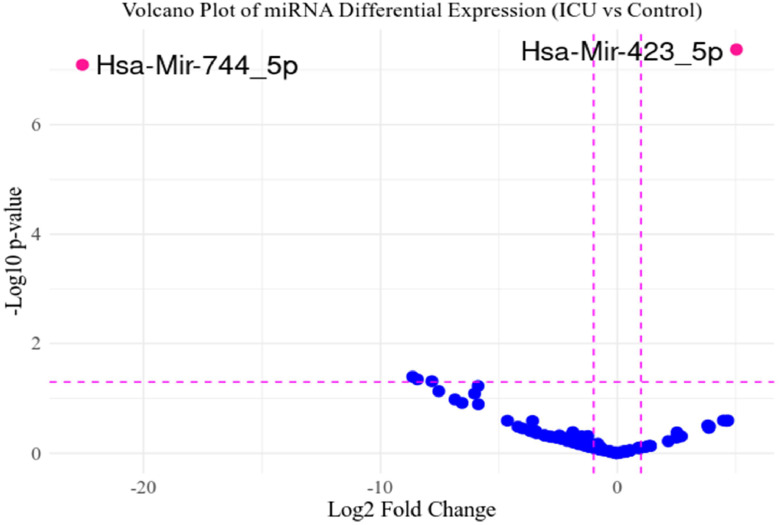
Volcano plot of ICU vs. Control group showing the relationship between fold change and statistical significance in miRNA gene expression. The X-axis represents the log2fold change, and the Y-axis represents the negative logarithm of the p-value (-log10). Higher values on this axis indicate more statistically significant results.

Differential expression analysis of ICU COVID-19 patients’ vs. healthy controls revealed 102 differentially expressed microRNAs. Considering adjusted p-value < 0.05 to be significant, two microRNAs, i.e., miR-423–5p and miR-744–5p, were observed to be most significantly differentially expressed with adjusted p-value = 4.12E-06 in both. Fold change ≥ 1.5 was considered significant. According to positive and negative log2 fold change values, it was evident that miR-423–5p was significantly upregulated, whereas miR-744–5p was significantly downregulated, respectively. These can also be visualized in the given heat map and volcano plot (Fig. 4 and 5).

The following heat map (Fig. 4) clearly shows all the upregulated and downregulated miRNAs of this group. The most significantly upregulated miR-423–5p is visible at the top with red color, while the most significantly downregulated miR-744–5p is present at the bottom with blue color. Whereas the volcano plot in Fig. 5 depicts the statistical difference between the differential expression of all miRNAs, and statistically significant miRNAs are clearly visible in it with a dark pink color.

### Ward vs. control

After the differential expression analysis between ward patients and healthy controls, a total of 91 miRNAs were found to be differentially expressed. Considering the FDR-adjusted p-value < 0.05 and fold change ≥ 1.5 was considered statistically significant, only one miRNA miR-486–5p was observed to be significantly DE with an adjusted p-value = 0.00501272398. A positive log2 fold change value of 10.09 clearly reflected that this miRNA was significantly upregulated.

**Fig. 6:**
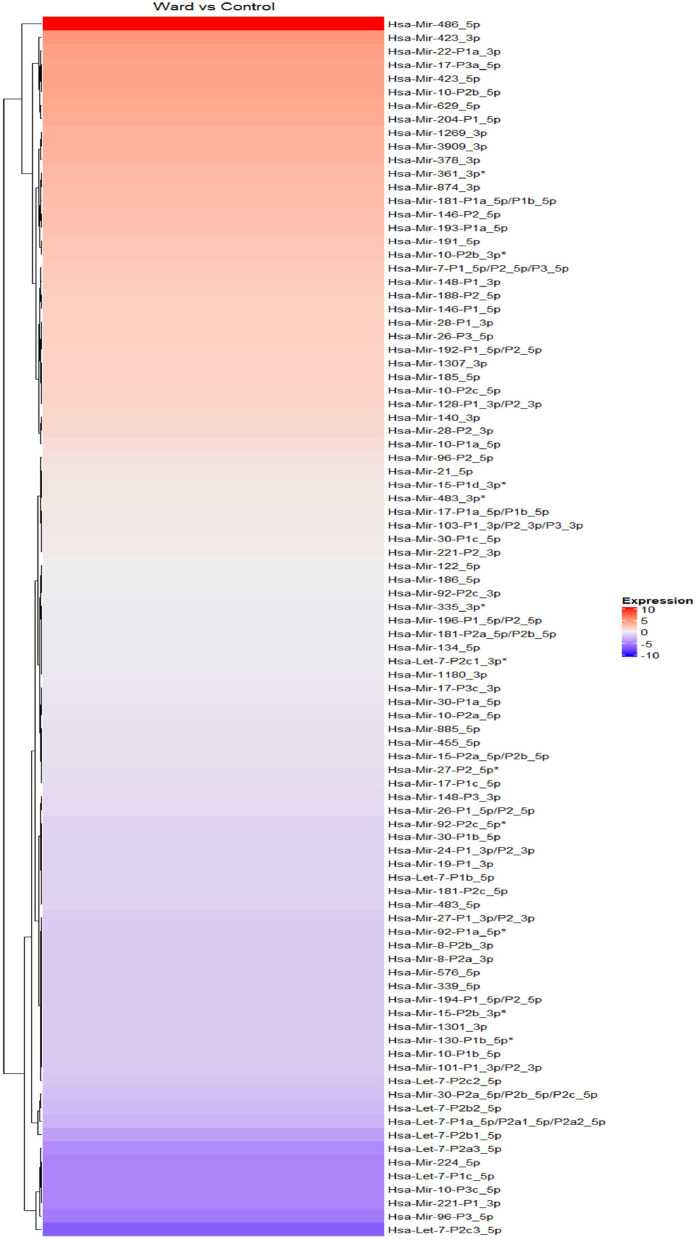
Ward vs. Control group Heat map showing all the DE host miRNAs in response to SARS-CoV-2 infection. The upregulated ones are shown in red, while the downregulated ones are in blue.

**Fig. 7:**
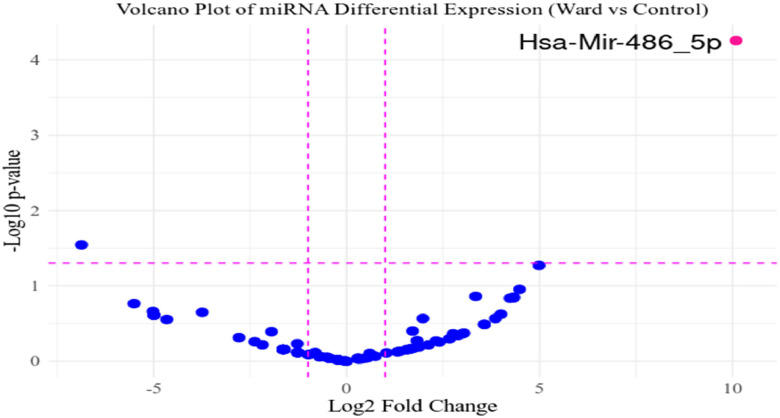
Volcano plot of Ward vs. Control group showing the relationship between fold change and statistical significance in their miRNA gene expression. The X-axis represents the log2fold change, and the Y-axis represents the negative logarithm of the p-value (-log10). Higher values on this axis indicate more statistically significant results.

The following heat map (Fig. 6) clearly shows all the upregulated and downregulated miRNAs of this group. The most significantly upregulated miR-486–5p is visible at the top with red color, while the downregulated miRNAs are present at the bottom with blue color. Whereas, the volcano plot in Fig. 7 depicts the statistical difference between the differential expression of all miRNAs, and a statistically significant miRNA is clearly visible in it with a dark pink color.

### Reported gene targets and their potential cellular pathways

Target Scan was used to explore the gene targets of DE miRNAs. The gene targets identified by Target Scan were neither found to be significant nor did they have any relation with the pathogenesis or intensity of the disease. Alternatively, a literature search revealed a number of significant gene targets for these three miRNAs that were found to be differentially expressed in the present study. The reported gene targets of these miRNAs, along with their cellular pathways closely linked to the underlying biochemical changes occurring in SARS-CoV-2 infection, are presented in Table 2.

**Fig. 8:**
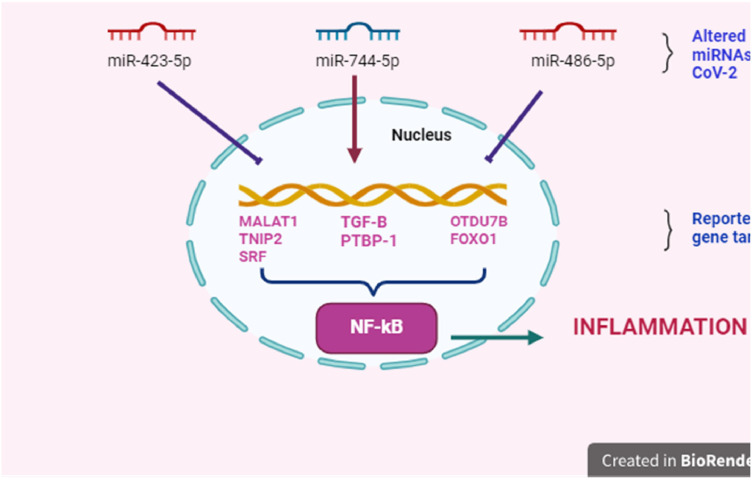
DE host miRNAs in SARS-CoV-2 infection. Upregulated miRNAs are shown in red and downregulated in blue. Literature based gene targets are visible on DNA and are shown to be inhibited by the upregulated miRNAs and stimulated by the downregulated ones. Collectively, all these effects may activate the NF-κB pathway that increases cytokine production, leading to inflammation.

It is evident from the reported gene targets of the three DE miRNAs that NF-κB is one of the major pathways that is altered by their regulation, as depicted in Fig. 8. This may further explain the inflammatory cascade initiated through NF-κB that culminates in cytokine elevation, which is a hallmark of COVID-19. Targeting the DE miRNAs responsible for acute inflammation may serve to prevent the vicious cytokine storm and thus ARDS, providing new therapeutic and prognostic avenues.

## Discussion

Inflammation is a key feature of the host immune response generated after exposure to SARS-CoV-2. It is characterized by a severe hyperinflammatory state reflected by a marked elevation in circulatory pro-inflammatory cytokines that may lead to acute respiratory distress syndrome ARDS.^30^ Research suggests that the cytokine storm in COVID-19 reflects rapid clinical deterioration and is strongly associated with the severity and poor prognosis of the disease.^31^ Circulating host miRNAs have been proposed as diagnostic and prognostic biomarkers of COVID-19,^13^ and their involvement in the development of the cytokine storm is documented in numerous studies.^15^^,^^32^

Biochemical evaluation is the key to effective assessment and management of SARS-CoV-2 infection. Assessment of biochemical markers revealed raised renal, hepatic, and cardiac function markers in both disease groups, with a greater elevation seen in ICU patients. Derangement in LFTs was limited to a significant increase in transaminases ALT and AST only. These results were in accordance with the previous literature that reports frequent mild elevations in COVID-19 patients that peak during their hospital stay and may persist even after discharge.^33^ Increased levels of BUN and creatinine in both disease groups, with a greater rise in ICU patients, reflect a deranged renal function that is in proportion to the intensity of the disease. The presence of ACE2 receptors specific for SARS-CoV-2 in renal tubules, along with a simultaneous increase in the circulatory inflammatory cytokines, are the factors that compromise renal function.^34^

All the inflammatory markers, including IL-6, CRP, LDH, and Ferritin, were significantly elevated in proportion to the severity of the disease. These observations were in line with the documented cytokine storm in COVID-19 that is responsible for this substantial increase in all these inflammatory markers.^35^ Ferritin had lesser but substantial clinical significance (p = 0.006) with respect to IL-6, CRP, and LDH in predicting the disease severity (p < 0.001). Such an observation was also made by Pore et al.^36^

A complete blood count has been a strong, cost-effective test to diagnose and assess the severity of COVID-19. Low Lymphocyte percentages in these patients, as observed in this study, have been strongly linked to the presence of SARS-CoV-2 infection. Moreover, high leucocyte count, high neutrophils, and low hemoglobin, as shown in the present study, are considered to be poor prognostic factors.^37^

A unique observation regarding d-Dimer was noted in this study. d-dimer levels, although found to be increased in both disease groups, were observed to be acutely raised in ward patients compared to the ICU ones. This finding reflects an intensely hyper-coagulable state of ward patients. In addition, d-dimer levels are strongly associated with disease progression and poor prognosis in COVID-19 patients.^38^ A lower rise in the d-dimer levels of ICU patients might suggest effective medical management of these patients to prevent any coagulation complications. An alternative explanation may be that the ICU patients may have had early mortality, so the survivors (who were sampled) had lower d-dimer levels, reflecting a classic survivor bias.

The current study also evaluated the Differential Expression (DE) of host circulatory miRNAs in severe (ICU) and moderate (ward) patients of SARS-CoV-2 infection. The miRNA findings are derived from a very small, underpowered cohort and are strictly preliminary in nature. These results serve only to generate hypotheses for future, properly powered studies. Moreover, the following molecular pathways are proposed on the basis of external literature and not demonstrated in this study.

The DE between the ICU and control group revealed 102 DE miRNAs, of which two had prime significance. miR-423–5p was highly upregulated, while miR-744–5p was significantly downregulated. The upregulation and involvement of miR-423–5p in the pathogenesis of COVID-19-related immune reactions have been reported recently.^14^ Interestingly, miR-423–5p was observed to be highly upregulated in lung epithelial cells of COVID-19 patients and promoted inflammation.^39^ Furthermore, miR-423–5p was shown to augment the NF-κB pathway by regulating MALAT-1, a long noncoding RNA that causes lung injury leading to ARDS, along with targeting TNFAIP3 Interacting Protein 2 (TNIP2), which is a known inhibitor of this pathway.^40^^,^^41^ The NF-κB pathway is specifically responsible for the elevation of pro-inflammatory mediators, including interleukins, which are implicated in the development of cytokine storm and exacerbation of SARS-CoV-2 infection.^42^ Recent work has identified SRF (serum response factor), another gene target of miR-423–5p^43^ An inverse relationship has been implied between SRF and the NF-κB pathway.^44^ Based on the literature, the authors hypothesize that miR-423–5p may contribute to COVID-19 severity by regulating the NF-κB pathway through its targets SRF, MALAT-1, and TNIP2.

Alternatively, miR-744–5p was observed to be significantly downregulated in ICU patients when compared with the healthy controls. Downregulation of miR-744–5p has been reported to upregulate Polypyrimidine Tract Binding Protein-1 (PTBP1), which is associated with pulmonary fibrosis.^45^ Moreover, PTBP1, through the alternate splicing of genes, may cause NF-κB activation as well.^46^ A significant inverse relationship was also reported between Human Leucocyte Antigen class G (HLA-G), an immunosuppressive molecule, and miR-744–5p expression.^47^ Thus, the downregulation of miR-744–5p in acutely affected COVID-19 patients may affect the immune response by increasing the HLA-G expression. Additionally, recent research indicates that this miRNA can suppress the MAPK signaling pathway by down-regulating Transforming Growth Factor Beta-1 (TGF-β1), which is a major cytokine known to regulate immune reactions, and its elevation leads to fibrosis in severe COVID-19 patients.^48^, ^49^, ^50^ The role of TGF-β1 may be explored as a therapeutic target that can help ameliorate the post-COVID complications in these patients.

Differential expression between COVID-19 ward patients and healthy controls of the current study manifested 91 differentially expressed miRNAs, including miR-486–5p, which was most significantly upregulated. A noteworthy finding in a study relates that the increased expression of miR-486–5p is linked with promoting acute lung injury/ARDS through an inflammatory response generated by targeting the OTU De Ubiquitinase 7B enzyme (OTUD7B) gene.^51^ This gene is reported to regulate the NF-κB pathway as well. If targeted, the inhibition of the OTUD7B gene can stimulate this pathway.^52^ This miRNA has been documented to be upregulated in severe patients of COVID-19, and it contributes to the pathogenesis of the disease by repressing OTUD7B and aggravating the inflammatory response.^53^

Forkhead Box O (FOXO) 1, a transcription factor, regulates the expression of various pro-inflammatory cytokines like IL-1β, TNF-α, TLR1, TLR4, and chemokine receptors and is also involved in T-cell homeostasis and adaptive immune response as well.^54^ Contemporary research in this regard has proposed an effective therapeutic approach based on activating FOXO1 to alleviate the inflammatory burst and combat SARS-CoV-2 infection.^55^ Intriguingly, FOXO1 is a documented gene target of miR-486–5p in human lung epithelial cells.^56^ It may be inferred from the given literature that elevated miR-486–5p expression in the moderate form of COVID-19 patients (ward) may downregulate FOXO1 and exacerbate the disease.

In spite of all research undertaken till now, the implementation of miRNAs as theragnostic tools hasn’t materialized yet. The regulatory mechanism through which these miRNAs affect signaling pathways specially the pro-inflammatory NF-κB pathway that significantly contributes to the severity and lethality of SARS-CoV-2, remains unclear. It was predicted earlier that if the links between the transcriptome and pro-inflammatory mediators in general and NF-κB in particular are well established, targeting them might be the best possible approach to alleviate the adverse outcomes of the disease. In addition, miRNA mimics and miRNA inhibitors may be used to restore the function of downregulated and upregulated miRNAs, reducing their pathogenic effects. Lastly, identification of DE miRNA profiles helps in predicting disease progression, outcomes, and response to therapy.^57^

Overall, the biochemical analysis manifests multiple organ dysfunction and hematological derangements in accordance with the disease status. These biochemical markers have a crucial prognostic role in the course of COVID-19. It must be noted that the clinical findings of this project come from a larger cohort, whereas the miRNA profiling by NGS has been performed on a small subset only. miRNA profiling by NGS indicated three miRNAs that were differentially expressed. miR-423–5p was upregulated and miR-744–5p was observed to be downregulated in the ICU vs. the control group. Whereas miR-486–5p was upregulated in the Ward Vs Control group. The molecular pathways related to the documented gene targets of these miRNAs were thoroughly studied. This metabolic pathway exploration revealed some significant details about the signaling pathways triggered in response to SARS-CoV-2 infection. It was intriguing to note that the diversified signaling pathways related to different gene targets seem to converge on the stimulation of the NF-κB pathway. Increased stimulation of this pathway plays a critical role in the production of numerous pro-inflammatory cytokines that serve as a prerequisite in the pathogenesis of COVID-19. It may be suggested that the inhibition of this pathway may go a long way in ameliorating the acute inflammatory state of these patients, thus improving the prognosis of the disease to a greater extent.

Conclusions drawn on the basis of documented research between DE miRNAs and their specified gene targets and molecular pathways also need further validation. Although the present study highlights differential microRNA expression alongside biochemical markers, the authors acknowledge the lack of correlation with clinical parameters such as cytokine levels and patient outcomes. Due to dataset limitations, multivariate analysis could not be performed. Future prospective studies with integrated clinical and molecular data are needed to validate these microRNAs as prognostic tools. Owing to financial and administrative constraints, the patient population was restricted to one city and the study was cross-sectional. This creates a generalizability bias. The miRNA findings are hypothesis-generating only and require further transcriptomic studies with a larger, independent, prospective, and well characterized clinical cohort followed by qPCR validation. This must also include integration and correlation of both biochemical and molecular data to get meaningful results. Additionally, clinical trials involving the use of miRNA mimics, miRNA inhibitors, molecular docking, or antisense oligonucleotides, as proposed recently for COVID-19 patients with varied disease status, is the way forward to assess their theragnostic potential.^58^

## Conclusion

Cytokine storm leads to an acute inflammatory state in patients afflicted with SARS-CoV-2 infection. Biochemical assessment reveals marked elevation in pro-inflammatory cytokines and biomarkers that lead to an intense state of inflammation. Significant rise in the levels of renal, hepatic and cardiac markers was proportionate with the intensity of the disease. miRNA profiling by NGS identified three DE miRNAs in these patients. It was explored that their documented gene targets cause the stimulation of the NF-κB pathway, which is of prime significance in developing this hyper-inflammatory state. These DE miRNAs, their gene targets, and pathway mediators leading to NF-κB activation are all suggested as potential prognostic and therapeutic biomarkers of this viral disease. Future larger cohort studies with integrated biochemical and miRNA profiling, their validation by qPCR, and clinical trials using miRNA mimics and inhibitors will ensure implementation of miRNAs as theragnostic tools for COVID-19.

## Ethics approval statement

The study was approved by the Institute of Molecular Biology and Biotechnology (IMBB) Ethical Review Committee, University of Lahore (IMBB/BBBC/22/120). All the data acquired was kept confidential.

## Informed consent statement

Written informed consent has been obtained from the patients to participate in this study and publish this paper.

## Data availability

Raw data files have been submitted to NCBI, SRA database project accession no: SUB14420173.

## Authors’ contributions

Mahnoor Khan: Conceptualization; data curation; writing original draft.

Awais Altaf: Methodology; supervision; writing review and editing.

Naeem Ashraf: Methodology; formal analysis; validation.

Syed Zeeshan Haider Naqvi: Project administration and supervision.

Hafiz Muhammad Hammad: Software; formal analysis; visualization.

Tahir Maqbool: Investigation; resources.

Tariq Aziz: Writing review and editing; supervision; resources; funding acquisition

Maha Abdullah Alwaili: Writing review and editing.

Rania Ali El Hadi Mohamed: Resources; validation.

Maher S. Alwethaynani: Visualization; software.

Fakhria A. Al-Joufi: Investigation.

Deema Fallatah: Validation; writing review and editing.

## Declaration of competing interest

The authors declare no conflicts of interest.

## Appendix Supplementary materials

Image, application 1

Image, application 2
