# Supplementary material for: Biochemical insights and microRNA profiling via next-generation sequencing in moderate and severe COVID-19 cases
Source: Clinics (Sao Paulo). 2026 Jun 10;81:101018. doi: 10.1016/j.clinsp.2026.101018 (PMC13273218; doi:10.1016/j.clinsp.2026.101018)
Supplement: Supplementary file 1 [file mmc1.docx]

CLINICS-D-25-01025_Supplementary Material

**Supplementary Table S1 Pre-sequencing RNA Quality Metrics of All Samples.**

| **Customer ID** | **Dante ID** | **Material Species** | **Concentration**  **(ng/μL)** | **RQN** | **Entry QC passed** |
| --- | --- | --- | --- | --- | --- |
| PKDTAAAA9824 | 105571-001-001 | Blood | 3.31 | 1 | yes |
| PKDTAAAA9815 | 105571-001-002 | Blood | 0.27 | 4.3 | yes |
| PKDTAAAA9806 | 105571-001-003 | Blood | 7.35 | 1.5 | yes |
| PKDTAAAA0790 | 105571-001-004 | Blood | 3.55 | 1.5 | yes |
| PKDTAAAA9781 | 105571-001-005 | Blood | 0.62 | 2.6 | yes |
| PKDTAAAA9772 | 105571-001-006 | Blood | 13.09 | 1.5 | yes |
| PKDTAAAA9763 | 105571-001-007 | Blood | 14.5 | 1.2 | yes |
| PKDTAAAA9754 | 105571-001-008 | Blood | 37.79 | 1 | yes |
| PKDTAAAA9745 | 105571-001-009 | Blood | 3.4 | 1.4 | yes |
| PKDTAAAA9736 | 105571-001-010 | Blood | 0.08 | 2.7 | yes |
| PKDTAAAA9727 | 105571-001-011 | Blood | 0.76 | 1.3 | yes |
| PKDTAAAA9718 | 105571-001-012 | Blood | 0.17 | 3.8 | yes |
| PKDTAAAA9709 | 105571-001-013 | Blood | 0.07 | 9.2 | yes |
| PKDTAAAA9693 | 105571-001-014 | Blood | 0.18 | 3.4 | yes |
| PKDTAAAA9684 | 105571-001-015 | Blood | 0.98 | 3 | yes |
| PKDTAAAA9675 | 105571-001-016 | Blood | 0.42 | 2.2 | yes |
| PKDTAAAA9666 | 105571-001-017 | Blood | 0.27 | 1.1 | yes |
| PKDTAAAA9657 | 105571-001-018 | Blood | 0.35 | 2 | yes |
| PKDTAAAA9648 | 105571-001-019 | Blood | 0.31 | 2.1 | yes |
| PKDTAAAA9639 | 105571-001-020 | Blood | 0.04 | 9 | yes |
| PKDTAAAA9620 | 105571-001-021 | Blood | 0.04 | 8.7 | yes |

**Supplementary Table S2 miRNA Sequencing Run Metrics of Selected Samples.**

| **Customer ID** | **Dante ID** | **Molarity (nM)** | **Yield (Mb)** | **Total Clusters** | **Quality % ≥ Q30** |
| --- | --- | --- | --- | --- | --- |
| PKDTAAAA9806 | 105571-001-003 | 0.83 | 1278 | 4233044 | 82.50% |
| PKDTAAAA0790 | 105571-001-004 | 1.3 | 1663 | 5507397 | 85.50% |
| PKDTAAAA9763 | 105571-001-007 | 1.24 | 1405 | 4653869 | 84.50% |
| PKDTAAAA9754 | 105571-001-008 | 2.17 | 1238 | 4099755 | 82.50% |
| PKDTAAAA9718 | 105571-001-012 | 1.88 | 3996 | 13231899 | 85.00% |
| PKDTAAAA9693 | 105571-001-014 | 1.29 | 2494 | 8257869 | 86.00% |
| PKDTAAAA9684 | 105571-001-015 | 2.38 | 3116 | 10317932 | 85.00% |
| PKDTAAAA9675 | 105571-001-016 | 1.48 | 3840 | 12715572 | 82.50% |
| PKDTAAAA9639 | 105571-001-020 | 0.75 | 1016 | 3365365 | 84.00% |
| PKDTAAAA9620 | 105571-001-021 | 1.27 | 1517 | 5021860 | 85.00% |

**Supplementary Table S3 Bioinformatics Parameters Used for NGS.**

| **Step** | **Tool / Software** | **Reference / Database** | **Key Parameters / Options** |
| --- | --- | --- | --- |
| Quality check | FastQC | – | Default parameters |
| Adapter & quality trimming | Trimmomatic | – | -a TGGAATTCTCGGGTGCCAAGG -m 17 -q 20 |
| Read alignment | Hisat2 | human, GRCh38 | -v 1 -m 10 --best --strata |
| miRNA quantification | miRge 3.0 | miRBase v22 | Counted reads uniquely mapping to mature miRNA sequences |
| Differential expression | DESeq2 | Version  1.30.1 | fitType="parametric", betaPrior=FALSE, FDR < 0.05 |
| Normalization | DESeq2 | 1.30.1 | Median‑of‑ratios method (estimateSizeFactors) |
| Multiple testing correction | DESeq2 | 1.30.1 | Benjamini–Hochberg FDR |
